# HDF-Net: Hierarchical Dual-Branch Feature Extraction Fusion Network for Infrared and Visible Image Fusion

**DOI:** 10.3390/s25113411

**Published:** 2025-05-28

**Authors:** Yanghang Zhu, Mingsheng Huang, Yaohua Zhu, Jingyu Jiang, Yong Zhang

**Affiliations:** 1 University of Chinese Academy of Sciences, Beijing 100049, China; zhuyanghang22@mails.ucas.ac.cn (Y.Z.); huangmingsheng@mail.ustc.edu.cn (M.H.); zhuyaohua@mail.sitp.ac.cn (Y.Z.); jiangjingyu23@mails.ucas.ac.cn (J.J.); 2 Shanghai Institute of Technical Physics, Chinese Academy of Sciences, Shanghai 200083, China

**Keywords:** image fusion, feature decomposition, attention mechanism, multimodality

## Abstract

To enhance scene perception and comprehension, infrared and visible image fusion (IVIF) integrates complementary data from two modalities. However, many existing methods fail to explicitly separate modality-specific and modality-shared features, which compromises fusion quality. To surmount this constraint, we introduce a novel hierarchical dual-branch fusion (HDF-Net) network. The network decomposes the source images into low-frequency components, which capture shared structural information, and high-frequency components, which preserve modality-specific details. Remarkably, we propose a pin-wheel-convolutional transformer (PCT) module that integrates local convolutional processing with directional attention to improve low-frequency feature extraction, thereby enabling more robust global–local context modeling. We subsequently introduce a hierarchical feature refinement (HFR) block that adaptively integrates multiscale features using kernel-based attention and dilated convolutions, further improving fusion accuracy. Extensive experiments on four public IVIF datasets (MSRS, TNO, RoadScene, and M3FD) demonstrate the high competitiveness of HDF-Net against 12 state-of-the-art methods. On the RoadScene dataset, HDF-Net achieves top performance across six key metrics—EN, SD, AG, SF, SCD, and SSIM—surpassing the second-best method by 0.67%, 1.85%, 17.67%, 5.26%, 3.33%, and 1.01%, respectively. These findings verify the generalization and efficacy of HDF-Net in practical IVIF scenarios.

## 1. Introduction

Image fusion has risen as a crucial research field in multimodal visual perception, driven by the rapid advancement of computer vision technologies [1,2]. This field has a variety of applications, including remote sensing, medical imaging, autonomous driving, object detection, and surveillance [3]. Among these tasks, infrared and visible image fusion (IVIF) is particularly significant in challenging environments, such as those with low illumination, nighttime scenes, and adverse weather conditions [4]. Infrared images capture thermal radiation [5], highlighting salient targets that may not be visible in low-light conditions, while visible images offer richer textures and spatial details. Fusing these modalities enables the integration of thermal and structural information, enhancing application tasks, including object detection, action recognition, and segmentation [6].

Despite their complementarity, the imaging mechanisms and semantic content of IR and VIS images differ substantially, presenting a challenge for the effective extraction and integration of features. Throughout the fusion process, a significant challenge is the delicate balance between fine-grained details and background structures [7]. IR images often lack sharp textures and borders, whereas VIS images are prone to noise and illumination variations. Failure to balance these aspects can lead to fused images with insufficient detail clarity or compromised background consistency, directly impacting downstream applications’ performance. For instance, inadequate fusion in surveillance and autonomous driving scenarios may result in missed targets or misclassification, reducing system reliability and safety [8]. Despite the advancements in traditional image fusion methods [9], such as multiscale decomposition or handcrafted fusion rules, their adaptability to dynamic environments is limited by their reliance on predefined operations and fixed fusion strategies, frequently leading to the loss of critical information.

Recent advances in deep learning have considerably enhanced IVIF performance. Convolutional neural networks (CNNs) have demonstrated strong local feature extraction capabilities [10], while generative adversarial networks (GANs) utilize adversarial learning to improve visual realism [11,12]. However, CNN-based approaches are inherently constrained by their limited receptive fields, making them less effective in modeling long-range dependencies and cross-modal relationships [13,14]. GAN-based methods, although promising, often suffer from training instability and mode collapse, and require large-scale datasets [15,16]. More recently, transformer-based architectures have been introduced into IVIF due to their success in modeling long-range dependencies and global context [17,18]. Compared with CNNs, transformers offer globally consistent feature representations and better preserve fine details in complex scenes. Nevertheless, current transformer-based fusion methods face challenges such as high computational complexity and inefficient feature alignment, which limit their scalability and practical application [19].

Moreover, existing deep learning-based methods often fail to explicitly differentiate between modality-shared features (e.g., backgrounds, global structures) and modality-specific features (e.g., textures, contours). This lack of targeted feature separation may lead to overfusion of low-frequency content and suppression of high-frequency details, compromising the overall quality of the fused image [20,21,22]. Studies have demonstrated that low-frequency features are frequently shared between IR and VIS images, while high-frequency features are typically modality-specific [23,24].

In response to these challenges, we propose a novel fusion framework named HDF-Net (hierarchical dual-branch feature extraction fusion network), designed explicitly for IVIF tasks. HDF-Net adopts a dual-branch encoder structure to extract modality-shared and modality-specific features separately, followed by a unified decoder to reconstruct the fused image [25]. The low-frequency branch is designed to capture global contextual information common to both modalities, whereas the high-frequency branch concentrates on intricate structures and textures.

To enhance the extraction of low-frequency information, we propose the pinwheel-convolutional transformer (PCT) module [26], which integrates a multilayer lite transformer architecture with a pinwheel-shaped convolutional kernel. The PCT module fulfils two critical functions. Firstly, employing asymmetric convolutions with pinwheel-shaped kernels effectively enlarges the receptive field, enabling robust feature extraction from low-contrast regions and large-scale background structures. Secondly, the PCT module adopts a localized, lightweight attention mechanism to replace conventional global attention, significantly reducing computational complexity while maintaining the Transformer’s capacity for global context modeling. This dual design addresses the inherent limitations of traditional convolutional layers in capturing global structural information and mitigates the high computational overhead commonly associated with standard transformer-based fusion networks.

In the high-frequency branch, we incorporate an invertible neural network (INN) module [27], which performs reversible transformations to preserve fine details, such as edges and textures, during fusion. INN ensures lossless information flow, which is essential for generating high-fidelity outputs. Our proposed HDF-Net introduces the following significant contributions:

(1) We propose a hierarchical dual-branch fusion architecture that demonstrates exceptional performance in image fusion. This architecture effectively integrates infrared and visible images by separating them into low-frequency, shared features and high-frequency, modality-specific details, ensuring optimal fusion quality.
(2) We design a novel PCT module to extract robust low-frequency features, strengthening the network’s capability to obtain background and large-scale structural data. Additionally, we employ an INN module to retain modality-specific high-frequency details with high fidelity.
(3) We design a hierarchical feature refinement (HFR) module to refine fused features further. In contrast to conventional linear refinement, HFR incorporates kernel operations (KO) and dilated convolutions (DC) to enhance feature expression and facilitate robust multiscale fusion. By integrating KO and DC, representation learning becomes more flexible and effective across both frequency branches, thus improving the overall quality and stability of the fusion.
(4) Our method surpasses existing approaches in visual quality and quantitative performance, as demonstrated by extensive experiments conducted on various public IVIF benchmark datasets, including MSRS [28], TNO [29], RoadScene [30], and M3FD [31].

## 2. Related Work

### 2.1. CNN- and GAN-Based Methods for IVIF

IVIF has attracted growing interest recently due to its broad applicability in computer vision tasks. Compared to single-modality imaging, fusing infrared (IR) and visible (VIS) images enables more comprehensive scene understanding, thereby boosting the robustness and validity of tasks such as object detection, scene perception, and target tracking [9,32,33,34].

Researchers have widely adopted CNN-based methods for their effectiveness in extracting local features. Early work, such as DenseFuse [35], used an autoencoder (AE) framework with dense connections to mitigate feature loss and enhance representation flow. However, its reliance on predefined fusion rules limited adaptability. Subsequently, IFCNN [36] introduced a fully trainable end-to-end architecture that learned both feature extraction and fusion in a data-driven manner. PMGI [37] proposed balancing gradient and intensity preservation, effectively maintaining detail and contrast in the fused images. SuperFusion [38] further addressed misalignment issues in IR and VIS images by introducing a feature alignment mechanism into the CNN framework. DAF-Net [39] integrated Restormer blocks and invertible neural networks (INNs) to improve global semantic consistency, better balancing local texture preservation and global structure modeling.

Despite these advances, CNN-based fusion approaches encounter numerous inherent challenges [40]. Their limited receptive fields impede the modeling of long-range dependencies and global context. At the same time, the overfusion of shared features and the neglect of modality-specific details are frequently the result of their lack of frequency-aware mechanisms. Additionally, their architectural rigidity limits their adaptability to various scene types and cross-modal data characteristics.

GAN-based approaches, such as FusionGAN [41], introduced adversarial training to generate perceptually realistic results. GANMcC [42] and AttentionFGAN [43] enhanced this framework with multidiscriminator setups and attention-based modules. TarDAL [44] introduced target-aware discriminators to better guide the fusion process for downstream tasks. However, these methods remain highly data-dependent, suffer from training instability, and often provide limited control over the interpretability of fused features.

### 2.2. Transformer-Driven Approaches and Limitations

Introducing the transformer architecture [45,46]—initially for NLP—marked a turning point in global modeling. Vision transformer (ViT) [47] and swin transformer [48] adapted this paradigm for image-level tasks. Due to their self-attention mechanism, transformers can more effectively model cross-modal relationships and long-range dependencies than CNNs [49,50].

SwinFuse [51] adopted swin transformer blocks for IVIF, balancing salient thermal targets and texture-rich visible details. However, its windowed attention introduces fragmented context modeling, potentially missing global consistency. CoCoNet [52] introduced contrastive learning and multilevel supervision but relied on extensive sample pairs and suffered from high training costs. CDDFuse [53] proposed a correlation-driven decomposition mechanism using Transformer–CNN hybrids to decouple shared and specific modality features. While it effectively improved feature disentanglement, it introduced high computational cost and complex pipeline design. Still, it suffered from information loss in high-frequency regions due to its dependence on global pooling strategies. PDFusion [54] adopted progressive dual-branch transformer fusion. Although it improved cross-layer semantic alignment, the framework lacked explicit frequency domain separation and involved repeated feature interactions that increased memory consumption and latency.

Despite leveraging Transformer structures, most prior methods do not explicitly separate frequency-aware representations and their attention mechanisms are computationally heavy, especially on high-resolution images. Furthermore, cross-modal alignment remains an open problem due to insufficient frequency-specific guidance.

To address the unresolved limitations in existing IVIF approaches, we propose HDF-Net, a fusion framework designed to achieve efficient and frequency-aware feature integration. Unlike prior works such as CDDFuse and PDFusion, which rely on heavy attention structures or implicit feature decoupling, HDF-Net employs a hierarchical dual-branch design that explicitly separates low-frequency shared content from high-frequency modality-specific details. This architectural decoupling improves interpretability while reducing feature entanglement.

Additionally, we present a lightweight transformer-based module (PCT) that integrates asymmetric convolutional operations to improve global modeling efficacy without imposing a high computational cost. PCT enables enhanced global context encoding with an expanded receptive field while avoiding the complexity of conventional full-attention designs. Furthermore, a feature refinement module (HFR) incorporating dilated convolutions and kernel operations is implemented to enhance multiscale alignment and suppress redundant activations. HDF-Net resolves numerous constraints associated with contemporary transformer-based fusion techniques, such as inadequate frequency-level separation, excessive computational overhead, and deficient cross-modal consistency.

## 3. Methods

This section begins by outlining the overall workflow of HDF-Net, followed by a comprehensive description of each module. Subsequently, we present the loss functions used in the model. For clarity, we will refer to the low-frequency global features as “core features” and the high-frequency local features as “refined features” in the following discussion.

### 3.1. Overview

**Figure 1:**
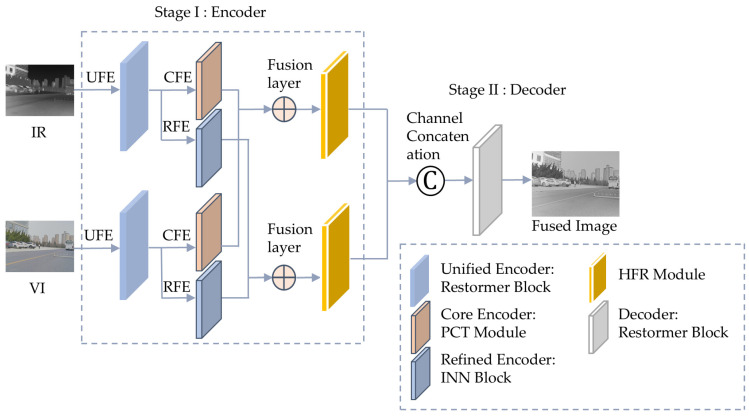
The overall architecture of HDF-Net. The encoder includes three main modules: a unified feature encoder (UFE), core feature encoder (CFE), and refined feature encoder (RFE), followed by a hierarchical feature refinement (HFR) module and a unified decoder.

HDF-Net comprises a dual-branch encoder responsible for feature extraction and segmentation, and a decoder that generates the fused image. Figure 1 illustrates the comprehensive infrastructure of HDF-Net.

### 3.2. Encoder Architecture

As shown in Figure 1, the encoder comprises four essential components: the unified feature encoder (UFE), the core feature encoder (CFE), the refined feature encoder (RFE), and the hierarchical feature refinement (HFR) module. We denote these components as $U\left(\cdot \right),C\left(\cdot \right),R\left(\cdot \right)$, and $H\left(\cdot \right)$, respectively. Each module plays a distinct role in extracting and refining multi-scale and modality-specific features, ensuring comprehensive feature representation.

#### 3.2.1. Unified Feature Encoder

The UFE is responsible for capturing supplementary and shared basic representations $\left\{\varphi_{I}^{U},\varphi_{V}^{U}\right\}$ from both infrared and visible images {I,V}, effectively extracting initial cross-modality information while preserving crucial structural details, i.e.,

(1) $$\varphi_{I}^{U}=U\left(I\right),\varphi_{V}^{U}=U\left(V\right).$$

We adopt the Restormer block in the UFE as a transformer-based encoder originally designed for image restoration. It applies self-attention in the frequency and channel domains, enabling efficient extraction of global features from high-resolution inputs while preserving computational efficiency [55].

#### 3.2.2. Core Feature Encoder

To obtain the core features $\left\{\varphi_{I}^{C},\varphi_{V}^{C}\right\}$ from the extracted shallow features $\left\{\varphi_{V}^{U},\varphi_{V}^{U}\right\}$, we define the transformation as:

(2) $$\;\varphi_{I}^{C}=C\left(\varphi_{I}^{C}\right),\varphi_{V}^{C}=C\left(\varphi_{V}^{U}\right).$$

To achieve this, we employ the pinwheel-convolutional transformer (PCT) module as the core feature encoder (CFE) core component, which enhances global and local feature representations.

**Figure 2:**
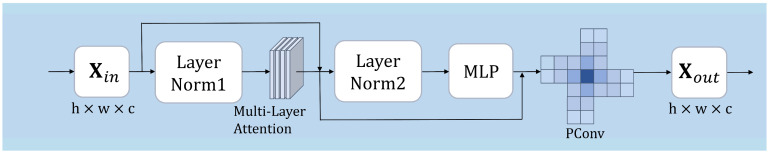
The architecture of the PCT module.

As illustrated in Figure 2, the PCT module integrates the multi-layer attention mechanism (MLA) and pinwheel-shaped convolution (PConv) to strengthen feature extraction. The MLA consists of multiple stacked self-attention layers, enabling effective modeling of long-range dependencies and feature refinement across various scales.

Given an input feature map $X\in R^{C\times H\times W}$, the MLA module implements hierarchical self-attention operations to simulate long-range dependencies. In the first layer, we define the initial attention computation as: $X_{res}^{\left(1\right)}=A_{1}\left(X\right),$ where $A_{1}\left(\cdot \right)$ represents the first AttentionBase module. The subsequent layers refine the attention output recursively:

(3) $$\;X_{res}^{\left(i\right)}=A_{i}\left(X_{res}^{\left(i-1\right)}\right),\forall i\in \left[2,L\right],$$

where L represents the total number of MLA layers. We apply a 1 × 1 convolution after the final attention layer to ensure consistency in feature dimensionality:

(4) $$X_{out}=W_{p}\times X_{res}^{L},$$

where $W_{p}$ represents a pointwise convolution, ensuring channel adaptation while maintaining the original spatial resolution.

We introduce pinwheel convolution (PConv) as a follow-up to the MLA module to refine the extracted features further. To enhance directional sensitivity and spatial diversity, we design the pinwheel convolution (PConv) module with four asymmetric padding strategies, denoted as: $P\left(1,0,0,3\right),P\left(0,3,0,1\right),P\left(0,1,3,0\right),P\left(3,0,1,0\right).$ Each padding tuple (top, right, bottom, left) in the pinwheel convolution (PConv) module defines an asymmetric spatial offset, shifting the receptive field off-center. This design enables the network to capture directional variations and spatial asymmetries—attributes often ignored by standard symmetric convolution. As demonstrated in prior work [26], such asymmetry aligns with the Gaussian-like distribution patterns commonly observed in infrared small targets, enhancing the feature extraction of weak signals; unlike conventional symmetric kernels, which aggregate features uniformly, our pinwheel-shaped kernels emphasize directional sensitivity, improving the detection of delicate structures and contour transitions in low-contrast or cluttered backgrounds. Mirroring the kernels would result in overlapping receptive fields, which dilutes feature diversity and hinders the network’s ability to model orientation-aware features.

After applying padding, we perform four parallel convolutions:

(5) $$\begin{array}{c} X_{1}^{\left(h^{\prime },w^{\prime },c^{\prime }\right)}=SiLU(BN(X_{P(1,0,0,3)}^{{(h}_{1},w_{1},c_{1})}⨂W_{2}^{\left(1,3,c^{\prime }\right)})),X_{2}^{\left(h^{\prime },w^{\prime },c^{\prime }\right)}=SiLU(BN(X_{P(0,3,0,1)}^{{(h}_{1},w_{1},c_{1})}⨂W_{2}^{\left(3,1,c^{\prime }\right)})),\\ X_{3}^{\left(h^{\prime },w^{\prime },c^{\prime }\right)}=SiLU(BN(X_{P(0,1,3,0)}^{{(h}_{1},w_{1},c_{1})}⨂W_{2}^{\left(1,3,c^{\prime }\right)})),X_{4}^{\left(h^{\prime },w^{\prime },c^{\prime }\right)}=SiLU(BN(X_{P(3,0,1,0)}^{{(h}_{1},w_{1},c_{1})}⨂W_{2}^{\left(3,1,c^{\prime }\right)})). \end{array}$$

The sigmoid linear unit (SiLU), also known as the Swish activation, is defined as SiLU(x)=x⋅sigmoid(x). It provides smooth activation and better convergence than ReLU. Batch normalization (BN) stabilizes and accelerates training by normalizing intermediate activations across each mini-batch. The convolution operation is denoted by ⨂, where each convolution kernel $W_{i}^{\left(k_{h},h_{w},c^{\prime }\right)}$ has a specific receptive field. For instance, $W_{1}^{\left(1,3,c^{\prime }\right)}$ represents a 1 × 3 convolution kernel with an output channel of $c^{\prime }$, capturing horizontal spatial dependencies. Where each convolution branch extracts different directional feature responses. We then concatenate these outputs along the channel dimension. Here, Cat refers to the channel-wise concatenation operator that merges the multi-branch outputs into a unified representation:

(6) $$\;X^{\prime \left(h^{\prime },w^{\prime },{4c}^{\prime }\right)}=Cat\left(X_{1}^{\left(h^{\prime },w^{\prime },c^{\prime }\right)},X_{2}^{\left(h^{\prime },w^{\prime },c^{\prime }\right)},X_{3}^{\left(h^{\prime },w^{\prime },c^{\prime }\right)},X_{4}^{\left(h^{\prime },w^{\prime },c^{\prime }\right)}\right).$$

Finally, the concatenated feature tensor undergoes normalization through a 2 × 2 convolution kernel. $W^{\left(2,2,c_{2}\right)}$ without additional padding. This operation refines the extracted features while maintaining spatial consistency. The dimensions of the output feature map are adjusted to predefined dimensions $h_{2}$ and $w_{2}$, ensuring seamless integration with standard convolutional layers. Moreover, PConv serves as an adaptive channel-attention mechanism by effectively analyzing the significance of different convolutional orientations. The final output representation $Y^{\left(h_{2},w_{2},c_{2}\right)}$ is computed as follows:

(7) $$Y^{\left(h_{2},w_{2},c_{2}\right)}=SiLU\left(BN\left(X^{\prime \left(h^{\prime },w^{\prime },{4c}^{\prime }\right)}⨂W^{\left(2,2,c_{2}\right)}\right)\right).$$

The final transformation adjusts the output spatial dimensions, setting the baseline, device control, and ending baseline while ensuring compatibility with standard convolutional layers. The effectiveness of PConv lies in its hierarchical multiscale receptive field, which mimics the Gaussian distribution of small target features.

#### 3.2.3. Refined Feature Encoder

Contrary to CFE, the RFE focuses on extracting high-frequency refined features from the unified features, expressed as:

(8) $$\varphi_{I}^{R}=R\left(\varphi_{I}^{U}\right),\varphi_{V}^{R}=R\left(\varphi_{V}^{U}\right).$$

Here, $R\left(\cdot \right)$ is implemented using an invertible neural network (INN) with affine coupling layers. The refined feature encoder (RFE) employs an INN-based architecture to preserve crucial edge and texture details for image fusion. The INN module guarantees lossless feature extraction by allowing the input and output features to be mutually reconstructable. Thus, we integrate INN blocks with affine coupling layers to preserve practical details.

#### 3.2.4. Fusion Layer

The fusion layer integrates the extracted core and refined features, maintaining consistency with the encoder’s feature extraction process, respectively, formulated as:

(9) $$\;\varphi^{C}=F_{C}\left(\varphi_{I}^{C},\varphi_{V}^{C}\right),\varphi^{R}=F_{R}\left(\varphi_{I}^{R},\varphi_{V}^{R}\right),$$

where $F_{C}$ and $F_{R}$ perform element-wise addition followed by pointwise convolution (1 × 1 Conv), representing linear transformations.

#### 3.2.5. HFR Module

We introduce the hierarchical feature refinement (HFR) module after obtaining the fused low-frequency and high-frequency features from the respective encoding branches. We further integrate multimodal information to ensure more effective interaction and representation of features.

(10) $$\;\varphi_{C}^{H}=H\left(\varphi^{C}\right),\varphi_{R}^{H}=H\left(\varphi^{R}\right).$$

**Figure 3:**
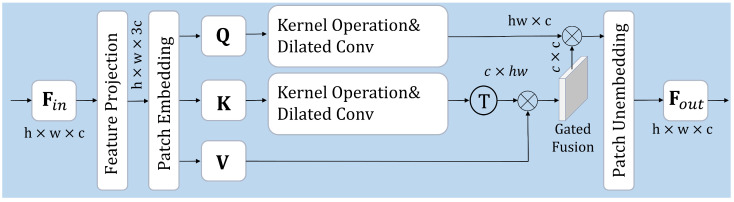
The architecture of the HFR module.

As is shown in Figure 3, an input feature map $F_{in}\in R^{h\times w\times c}$ undergoes a feature projection step that first transforms it into an expanded representation $F_{in}\in R^{h\times w\times 3c}$ to enhance channel-wise feature richness. Subsequently, a patch embedding operation is implemented to partition the input feature into nonoverlapping segments, producing the embedded representations $\;\{Q,K,V\}\in R^{h\times w\times c}$, which are then processed by the self-attention mechanism.

To improve feature representation robustness, we adopt a Mercer-based kernel operation to reconstruct Q and K through a projection mapping: $\tilde{Q}=M\left(Q\right),\tilde{K}=M(K)$. Next, dilated convolution (DC) is employed to process $Q^{\prime }$ and $K^{\prime }$: $Q^{\prime }=DC\left(\tilde{Q}\right),K^{\prime }=DC(\tilde{K})$. We then compute the attention mechanism as follows:

(11) $$Att\left(Q^{\prime },K^{\prime },V\right)=Q^{\prime }\cdot \sigma \left(K^{\prime T}\cdot V\right),$$

where σ(·) dynamically adjusts the attention weights to balance the contributions of different features, enabling adaptive fusion between core and refined features.

HFR integrates kernel operation (KO) with dilated convolution (DC) to construct a hierarchical representation space. KO maps the input features into a higher-dimensional space, allowing for more expressive similarity measurement between Q and K. Meanwhile, DC dynamically expands the receptive field while preserving spatial structures, effectively capturing multi-scale dependencies. The refined $\tilde{Q}$ and $\tilde{K}$ are then utilized to compute adaptive attention weights, followed by a gated fusion mechanism that combines these attention- weighted responses with the value (V) representation. 

### 3.3. Decoder

The decoder rebuilds the fused image by merging the core and refined features across the channel dimension:

(12) $$G_{out}=Dec\left(\varphi_{C}^{H},\varphi_{R}^{H}\right).$$

To ensure consistency with the unified feature encoder (UFE), the decoder employs a series of Restormer blocks as its core computational units. These blocks are configured with the same embedding dimensionality, attention heads, and feedforward structures as in UFE, facilitating effective feature reconstruction while preserving global context and local detail integrity. This design balances structural simplicity and representational fidelity, avoiding redundant computation while ensuring that cross-modal semantic information is effectively integrated into the final fused output.

### 3.4. Loss Function

Given the unavailability of ground truth in IVIF, conventional supervised learning methods are unavailable. To address this, we design an unsupervised loss function that encourages the preservation of shared low-frequency structures and modality-specific high-frequency details. We define the overall loss as:

(13) $$L_{total}=L_{rec}+{\lambda_{1}L}_{grad}+{\lambda_{2}L}_{decomp}+\lambda_{3}L_{feat}.$$

Here, the reconstruction loss $L_{rec}$ ensures that the fused image F remains structurally close to the most informative parts of both source images $I_{IR}$ and $I_{VI}$, by minimizing the intensity and structural similarity errors:

(14) $$\;L_{rec}={\left\|F-\max\left(I_{IR},I_{VI}\right)\right\|}_{1}+\mu \left(1-SSIM\left(F,\max\left(I_{IR},I_{VI}\right)\right)\right).$$

The max operator selects salient targets (e.g., warm bodies from IR and textures from VIS), and the SSIM term improves structural integrity by maintaining local luminance and contrast.

To enhance edge and texture information, especially in high-frequency regions extracted by the INN block, we introduce the gradient loss $L_{grad}$, which coordinates the gradients of the fused images and the input images:

(15) $$L_{grad}={\left\|\nabla F-\max\left(I_{IR},I_{VI}\right)\right\|}_{1},$$

$L_{grad}$ focuses on preserving edge and texture information, especially in high-frequency regions. It is critical for capturing contours (e.g., pedestrians, vehicles), especially when INN is applied for detail preservation. The Sobel operator ∇ detects image gradients, aligning spatial edges of the fused result with the sources.

The decomposition loss $L_{decomp}$ is designed to enforce a high correlation between core features while encouraging the refined features to remain distinct:

(16) $$L_{decomp}=\frac{{\left[CC(\varphi_{I}^{R},\varphi_{V}^{R})\right]}^{2}}{CC\left(\varphi_{I}^{C},\varphi_{V}^{C}\right)+\varepsilon },$$

where CC denotes the Pearson correlation coefficient, $\varphi^{C}$ and $\varphi^{R}$ represent core and refined features. The numerator promotes a high correlation between refined features (IR and VIS), encouraging shared structure. The denominator discourages excessive similarity in core features, enforcing that modality-specific features remain distinct. This balance avoids over-fusion and preserves modality-specific cues.

Moreover, considering that the HFR module refines feature representations through multiscale interactions, we further introduce a feature similarity loss $L_{feat}$, which ensures that the refined features $\varphi_{C}^{H}$ and $\varphi_{R}^{H}$ remain consistent with their original counterparts:

(17) $$L_{feat}={\left\|\varphi_{C}^{H}-\varphi^{B}\right\|}_{1}+{\left\|\varphi_{R}^{H}-\varphi^{D}\right\|}_{1}.$$

The HFR module generates enhanced representations via multiscale fusion. This term regulates feature drift, ensuring that:

(1) Refined high-level features (after HFR) remain consistent with their low-level counterparts (before HFR).
(2) It prevents oversmoothing or semantic deviation during refinement.

## 4. Results and Analysis

This section presents a systematic series of experiments conducted under various settings to validate the effectiveness and generalization capability of the proposed HDF-NET. First, we outline the experimental environment, which includes datasets, implementation details, and evaluation metrics. Secondly, we conduct comparative experiments against state-of-the-art fusion methods to showcase the superiority of HDF-NET in both qualitative and quantitative evaluations. Finally, we perform an ablation study to analyze the contributions of key components in our model and verify their impact on fusion performance.

### 4.1. Experimental Setup

#### 4.1.1. Datasets

In our IVIF experiments, we assess the proposed method on four widely recognized benchmark datasets: MSRS, TNO, RoadScene, and M3FD. The infrared images are captured using thermal infrared (LWIR) sensors operating primarily in the 8–14 µm wavelength range. We train the HDF-NET model on the MSRS training dataset, which comprises 1083 pairs of images from two modalities. For performance evaluation, we test the model on the MSRS test set (300 pairs), RoadScene (55 pairs), TNO (42 pairs), and M3FD test set (300 pairs). These datasets encompass diverse real-world scenarios, allowing us to assess the stability and versatility of the proposed method. We thoroughly evaluate the model’s ability to preserve structural details, enhance contrast, and produce high-quality fusion results across different datasets.

#### 4.1.2. Implement Details

We conducted all trials on a workstation equipped with an Intel(R) Core (TM) i7-14700KF CPU (Intel Corporation, Santa Clara, CA, USA) and an NVIDIA GeForce RTX 4070 Ti Super GPU (NVIDIA Corporation, Santa Clara, CA, USA). For preprocessing, we randomly crop the training images into 128 × 128 patches. The network undergoes training for 100 epochs in a single-stage setting employing a batch size of 16. We utilize the Adam optimizer, starting with a learning rate of $10^{\left(-4\right)}$, which decays by a factor of 0.5 every 20 epochs. The unified feature encoder (UFE) consists of four Restormer blocks, each utilizing eight attention heads and an embedding dimension of 64 for the network’s hyperparameter settings. The core transformer encoder (CTE) is constructed using lite transformer (LT) blocks that are dimensionally consistent with those used in the unified feature encoder (UFE), sharing the same embedding size and number of attention heads to maintain feature transformation compatibility. The loss function parameters used in Equation (13) are empirically set to $\lambda_{1}=1,\;\lambda_{2}=2,\;\lambda_{3}=1$, respectively, based on a grid search on the MSRS validation set. These values balance texture enhancement, modality decoupling, and feature consistency to ensure training stability and fusion quality. For RGB visible images (e.g., Figure 9), we convert them to the YCrCb color space, perform fusion on the Y (luminance) channel using our proposed method, and then recombine the original Cr and Cb channels with the fused Y to reconstruct the final color image.

#### 4.1.3. Metrics

The comparative analysis is conducted across multiple benchmark datasets. We evaluate the performance using a set of widely recognized quantitative metrics, including entropy (EN), standard deviation (SD), average gradient (AG), spatial frequency (SF), structural similarity index (SSIM), mutual information (MI), and sum of correlation of differences (SCD). These metrics comprehensively assess the fusion outcomes in preserving information, contrast enhancement, detail retention, and structural consistency. Since the IVIF task lacks a ground truth fused image, all evaluation metrics (e.g., SSIM, MI, SCD, EN) are computed between the fused image and each input source image (IR and VIS), following widely adopted practice in prior fusion studies [36,39,53].

### 4.2. Comparison Results

**Figure A1:**
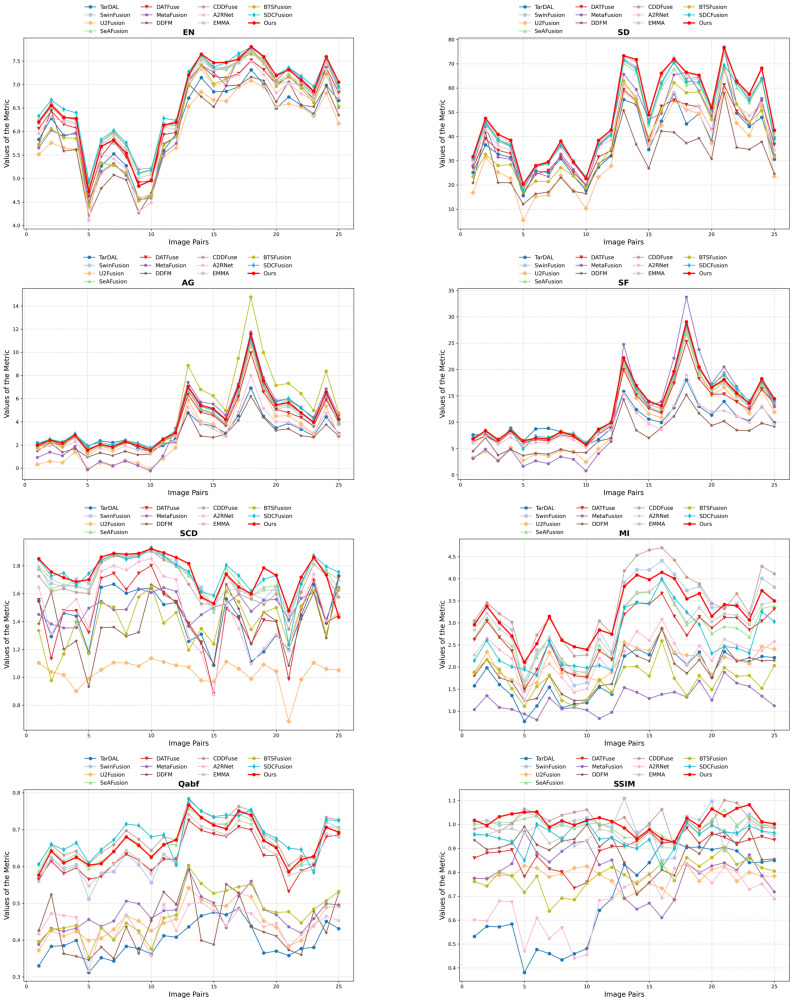
Quantitative comparison of HDF-NET with 12 state-of-the-art methods on 25 pairs of images in the MSRS dataset.

**Figure A2:**
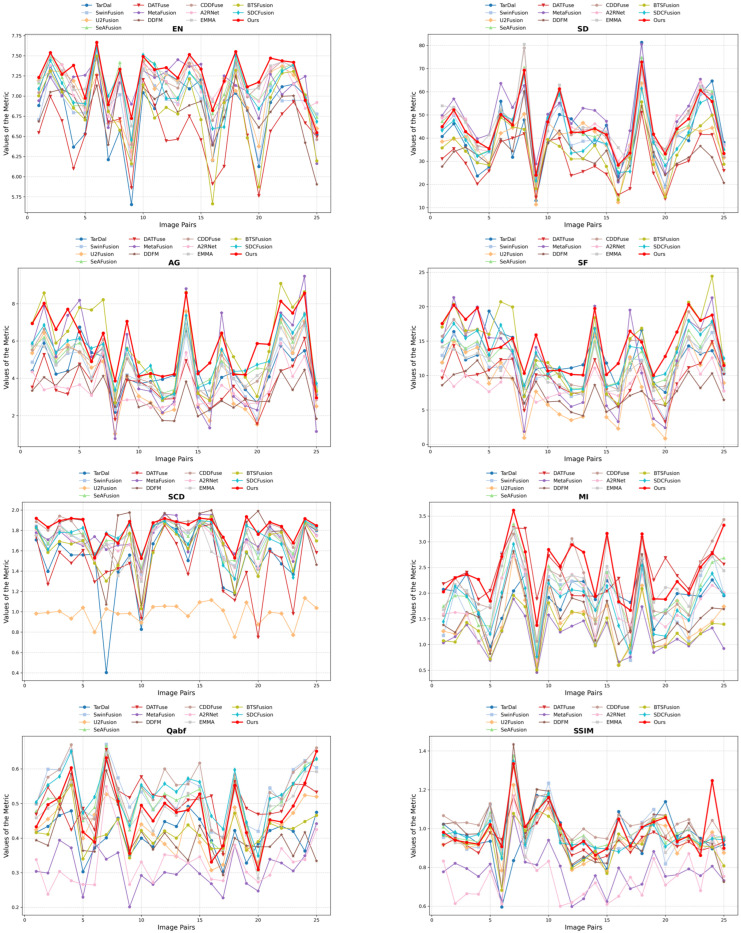
Quantitative comparison of HDF-NET with 12 state-of-the-art methods on 25 pairs of images in the TNO dataset.

**Figure A3:**
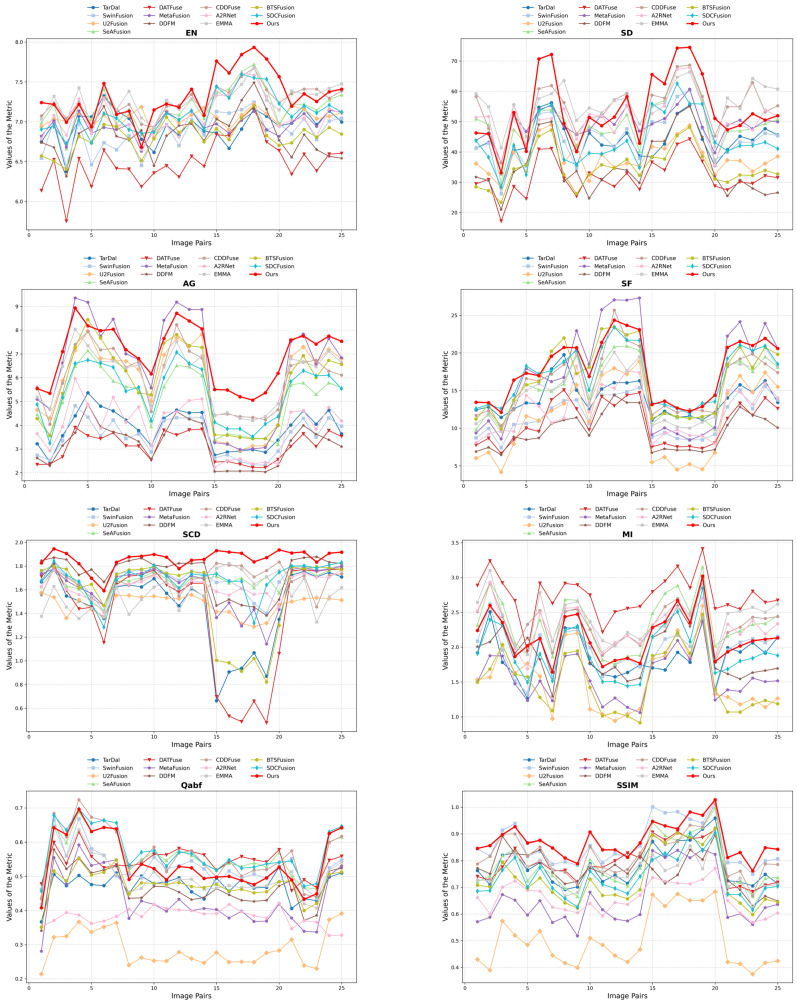
Quantitative comparison of HDF-NET with 12 state-of-the-art methods on 25 pairs of images in the RoadScene dataset.

**Figure A4:**
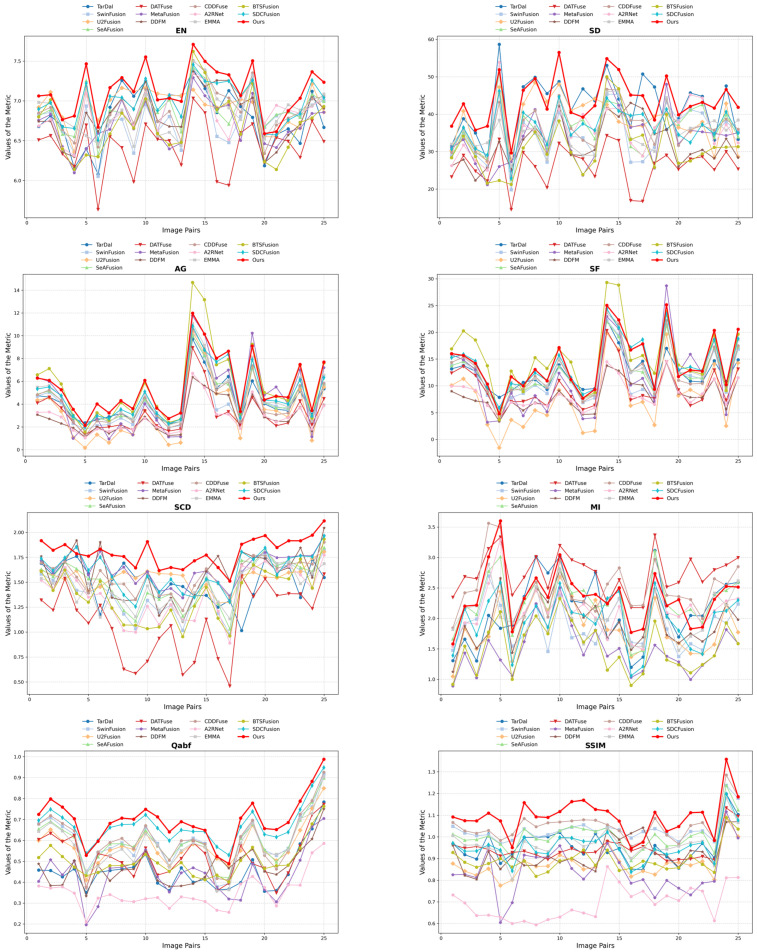
Quantitative comparison of HDF-NET with 12 state-of-the-art methods on 25 pairs of images in the M3FD dataset.

To thoroughly assess the effectiveness of our proposed HDF-NET, we perform extensive comparisons with 12 state-of-the-art (SOTA) image fusion methods through qualitative and quantitative analyses. The selected baseline methods include TarDAL [44], SwinFusion [56], U2Fusion [57], SeAFusion [58], DATFuse [59], MetaFusion [60], DDFM [61], CDDFuse [53], A2RNet [62], EMMA [63], BTSFusion [64], and SDCFusion [65]. To guarantee an impartial and consistent evaluation, we utilize the publicly available implementations of these methods and apply the same experimental settings, including adversarial conditions, hyperparameters, and input preprocessing procedures. Appendix A (Figure A1, Figure A2, Figure A3 and Figure A4) shows per-pair metric performance curves for a more detailed performance trajectory across each dataset.

#### 4.2.1. Results of the MSRS Dataset

**Figure 4:**
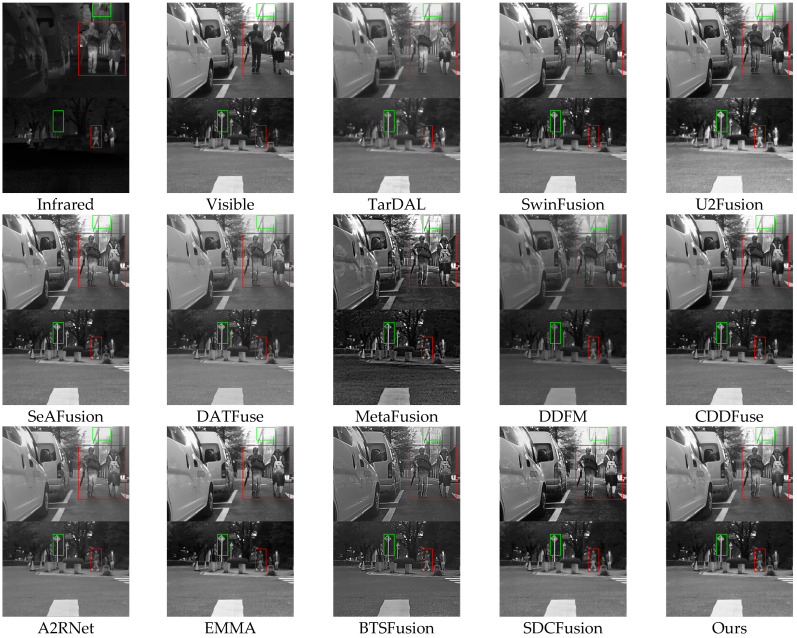
Qualitative comparison of HDF-Net with 12 state-of-the-art methods on the MSRS dataset.

Qualitative analysis: Figure 4 shows fused results from multiple methods on the MSRS dataset. In the first scene, models such as SwinFusion, U2Fusion, DATFuse, and BTSFusion suffer from blurred edges and loss of detail in pedestrians and building contours. A2RNet improves target clarity but fails to maintain background definition. TarDAL performs poorly overall, while MetaFusion enhances infrared intensity but introduces unnatural contrast artifacts. In contrast, HDF-Net delivers sharp foreground-background separation and precise texture details, attributed to its PCT module’s expanded receptive field and the HFR module’s effective cross-scale refinement.

**Figure A5:**
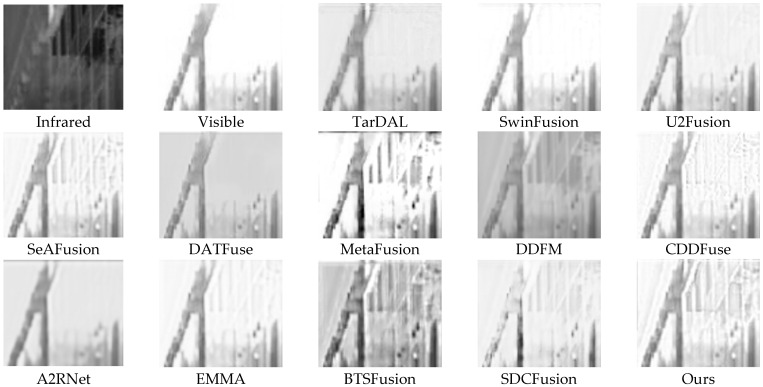
Qualitative close-up comparisons on a challenging scene from the MSRS dataset.

Appendix B (Figure A5) presents enlarged views of challenging regions for closer inspection. HDF-Net consistently maintains structural clarity and clean textures under intense lighting and low contrast, whereas other methods show blur or oversharpening. This highlights the robustness of our frequency-aware architecture in preserving fine textures and resisting illumination-induced degradation.

Quantitative analysis: Figure A1 compares HDF-NET and twelve advanced methods on 25 image pairs on the MSRS dataset, with average results summarized in Table 1. HDF-NET performs best in EN, SD, SF, SCD, and SSIM and ranks second in AG and MI. High EN and SF scores indicate rich information content and well-preserved textures, while superior SD reflects enhanced contrast. Leading SCD and SSIM values confirm strong structural preservation and similarity to source images, illustrating the robustness and overall capability of the proposed method.

#### 4.2.2. Results of the TNO Dataset

**Figure 5:**
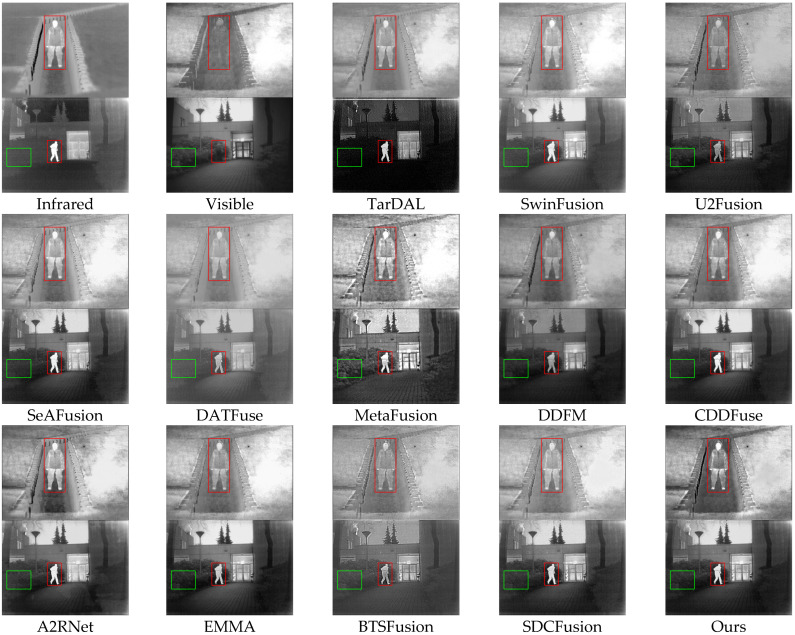
Qualitative comparison of HDF-Net with 12 state-of-the-art methods on the TNO dataset.

Qualitative analysis: Figure 5 shows results from TNO scenes, where traditional methods like TarDAL, SwinFusion, U2Fusion, and BTSFusion fail to preserve structural clarity under thermal and illumination discrepancies. TarDAL and U2Fusion produce blurred pedestrian figures, while SeAFusion struggles with vegetation structure preservation. A2RNet improves pedestrian representation but underperforms on background texture. EMMA and CDDFuse offer more precise contours, but minor texture degradation remains. However, HDF-NET preserves small-scale pedestrian features and large-scale background structures with high visibility. These results reflect the strength of HDF-NET in capturing modality-specific structures. Specifically, the INN module preserves edge details without introducing artifacts, while the PCT-enhanced CFE ensures global structure consistency under complex thermal-visual discrepancies.

Quantitative analysis: Figure A2 compares HDF-NET and twelve advanced fusion methods on the TNO dataset, with average results summarized in Table 2. HDF-NET achieves leading performance in EN, SF, SCD, and MI, ranking second in SD, AG, and SSIM. These results highlight HDF-NET’s robust design, particularly the dual-branch architecture and the HFR module, which ensures effective fusion across modalities even under varying thermal contrast and complex scenes.

#### 4.2.3. Results of the RoadScene Dataset

**Figure 6:**
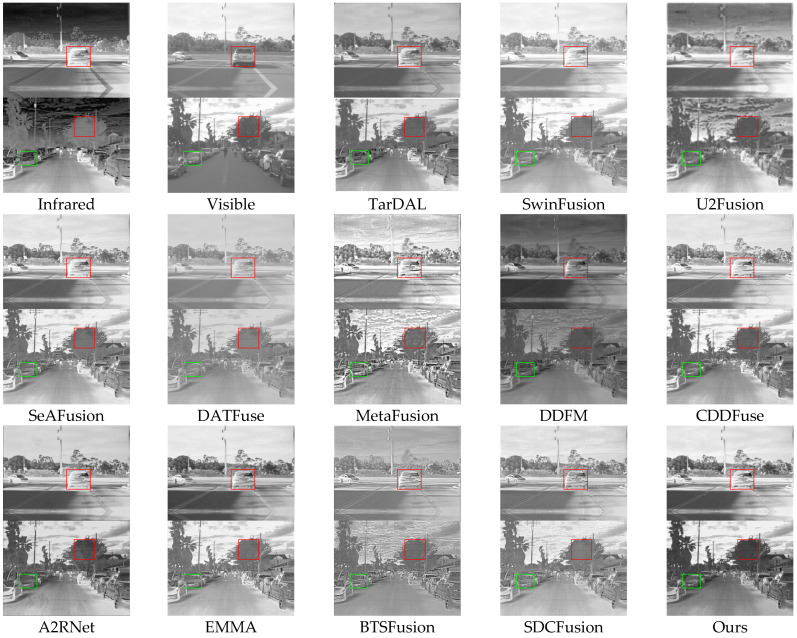
Qualitative comparison of HDF-Net with 12 state-of-the-art methods on the RoadScene dataset.

Qualitative analysis: Figure 6 illustrates representative fusion results in driving scenes. Several methods, including SwinFusion, DATFuse, DDFM, and BTSFusion, produce dark or low-contrast outputs, especially in regions containing vehicles, pedestrians, and roadside vegetation. TarDAL and U2Fusion blur critical object boundaries, while MetaFusion causes over-enhancement, introducing unrealistic visual artifacts. Although CDDFuse, A2RNet, and EMMA preserve more detail, they still struggle with fine texture in dense regions. HDF-NET consistently produces natural textures, well-defined contours, and high perceptual clarity. The HFR module plays a crucial role in these complex street scenes by adaptively integrating frequency-specific features, ensuring vehicles, pedestrians, and vegetation maintain clarity and contrast, which allows HDF-NET to deliver stable results in cluttered environments.

Quantitative analysis: Figure A3 compares HDF-NET with twelve advanced methods on the RoadScene dataset, with mean results in Table 3. Evaluated using eight standard metrics, HDF-NET ranks highest in EN, SD, AG, SF, SCD, and SSIM, confirming its robustness in diverse fusion scenarios. These results underscore the benefits of the lightweight PCT module and the hierarchical fusion strategy, which together yield more precise, more semantically consistent representations in complex road scenes.

#### 4.2.4. Results of the M3FD Dataset

**Figure 7:**
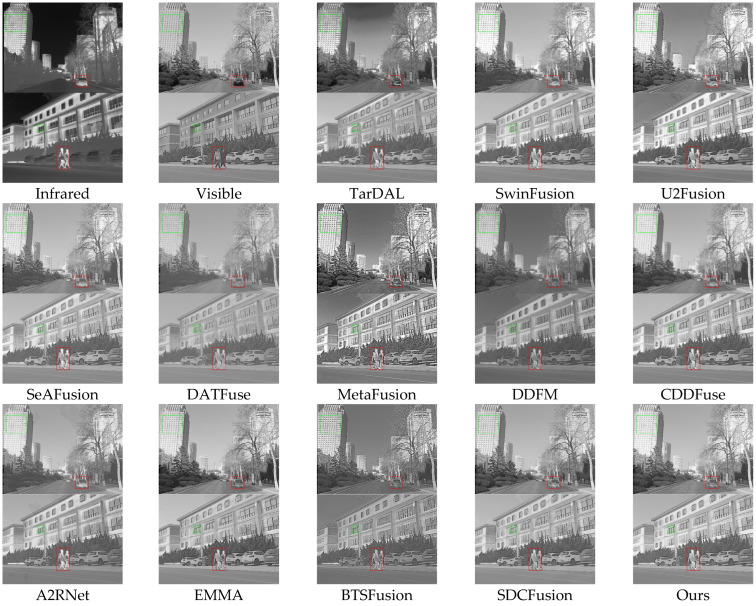
Qualitative comparison of HDF-Net with 12 state-of-the-art methods on the M3FD dataset.

Qualitative analysis: Figure 7 illustrates fused images produced by different methods across two representative scenes. TarDAL, U2Fusion, SeAFusion, BTSFusion, and A2RNet exhibit noticeable blurring, leading to indistinct pedestrian and architectural details. In Scene 1, SwinFusion, DATFuse, and DDFM suffer from inadequate brightness and contrast, resulting in distant targets blending into the background and obscuring structural features. MetaFusion displays excessive contrast and unnatural sharpness, particularly in building regions. While EMMA and CDDFuse effectively preserve pedestrian and vehicle details, they experience slight texture loss and background clarity issues. In contrast, HDF-NET delivers superior visual quality across all targets and environments, thanks to its efficient separation of frequency components and robust refinement across modalities.

Quantitative analysis: Figure A4 compares the proposed HDF-NET with twelve state-of-the-art fusion methods on the M3FD dataset, presenting the average results in Table 4. Evaluated using eight standard metrics, HDF-NET outperforms other methods, achieving top results in EN, SD, SCD, $Q^{AB/F}$, and SSIM. These scores confirm the method’s capability to integrate complementary infrared and visible information, capturing fine details while preserving visual consistency and demonstrating robustness and effectiveness in complex and dynamic scenes.

### 4.3. Ablation Study

To evaluate the validity of the pinwheel-convolutional transformer (PCT) module and the hierarchical feature refinement (HFR) module in image fusion tasks, we conduct a series of ablation studies on the MSRS dataset. We specifically design three experiments: (i) replacing the PCT module in the core feature encoder (CFE) with the standard lite transformer (LT) to examine the contributions of multilayer attention and pinwheel convolution to low-frequency feature extraction; (ii) removing the HRM module to analyze its role in multi-scale feature refinement and cross-modality alignment; and (iii) removing both PCT and HRM to form a minimal baseline for comparison.

As shown in Table 5, our full HDF-Net achieves top performance across all evaluation metrics, demonstrating the superiority of our proposed modules. Notably, removing the PCT module leads to degraded detail representation and contrast, highlighting its effectiveness in enhancing semantic low-frequency features. Omitting HRM causes significant drops in structural similarity metrics (SCD and SSIM), indicating its contribution to preserving spatial coherence and enhancing global-local feature interactions. The joint removal of both modules results in the worst performance, underscoring their complementary benefits.

**Figure 8:**
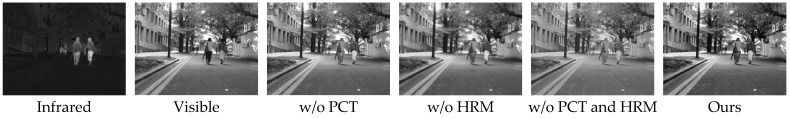
Visual comparison of ablation study results for each module on the MSRS dataset.

Figure 8 illustrates visual comparisons under different ablation settings. Incorporating the HRM module enhances the preservation of delicate structures, resulting in more precise textures of trees and sidewalk edges and improving global-local consistency. The PCT module notably enhances image contrast and dynamic range, making thermal targets (e.g., pedestrians) more distinguishable and seamlessly integrated with the background under complex illumination conditions. Combining HRM and PCT, the complete HDF-Net achieves an optimal balance between thermal target enhancement and visible detail preservation, demonstrating robustness and effectiveness in real-world fusion scenarios.

### 4.4. Medical Image Fusion

We performed a medical image fusion (MIF) test of 100 pairs of PET-CT and MRI-PET images. All models were initially trained on the IVIF projects and directly generalized to the MIF projects. The evaluation metrics are consistent with those used in the IVIF setting, aligning with state-of-the-art methods.

**Figure 9:**
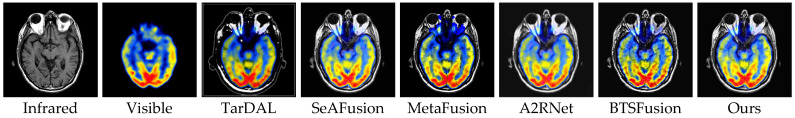
Visual comparison with 5 state-of-the-art methods for the MIF task.

As shown in Figure 9, our method achieves visually and quantitatively superior performance. In particular, it demonstrates an enhanced capacity to preserve anatomical structures from MRI and metabolic details from PET, yielding fused images with more explicit tissue boundaries and better representation of functional intensity. Compared with SOTA methods such as SeAFusion, MetaFusion, and BTSFusion, our approach integrates cross-modal features more effectively, achieving a more balanced and informative fusion result across different MIF modalities.

### 4.5. Discussion

This section has demonstrated the superior performance of HDF-Net across multiple standard benchmarks, including MSRS, TNO, RoadScene, and M3FD. The proposed network consistently achieves leading results in key objective metrics such as EN, SD, AG, SF, SCD, and SSIM, validating its effectiveness in preserving structural details, enhancing salient targets, and maintaining global contrast under diverse imaging conditions.

While HDF-Net exhibits overall robustness, certain limitations persist. Preserving fine-grained textures can remain suboptimal in scenarios involving extremely low-contrast illumination or highly saturated infrared signals (e.g., intense thermal reflections at night). These observations provide meaningful guidance for the future extension and deployment of HDF-Net in broader fusion scenarios.

## 5. Conclusions

This paper presents HDF-Net, a hierarchical dual-branch network designed explicitly for fusing infrared and visible images. HDF-Net addresses the longstanding trade-off between structural integrity and texture detail preservation in fusion tasks by explicitly modeling modality-shared core and modality-specific refined features through a dedicated dual-branch encoder. To further enhance global contextual representation, particularly in low-contrast regions, we propose the pinwheel-convolutional transformer (PCT) module, which integrates multi-head attention with directional pinwheel convolution to expand the receptive field significantly. In addition, the hierarchical feature refinement (HFR) module leverages Mercer kernel mapping and dilated convolutions to construct a robust multi-scale attention mechanism, enabling more adaptive and discriminative cross-modal feature integration.

Comprehensive experiments on four publicly accessible IVIF datasets prove that HDF-Net consistently outperforms 12 state-of-the-art fusion approaches across eight quantitative evaluation metrics, yielding superior information preservation and structural fidelity results. Furthermore, we broaden the scope of HDF-Net to medical image fusion tasks such as MRI-PET, validating its strong generalization capabilities and practical utility beyond conventional fusion scenarios. In upcoming research, we aim to explore further the deployment of HDF-Net in broader fusion contexts, including multi-exposure image fusion and night vision enhancement, as well as its potential to improve performance in downstream vision projects, such as object recognition, instance segmentation, and scene comprehension.

## Figures and Tables

**Table 1:** Quantitative results of comparative experiments on the MSRS dataset. The highest values are presented in **bold** to highlight the comparative performance, while the second-highest values are underlined.

Dataset: MSRS Fusion Dataset								
Method	EN	SD	AG	SF	SCD	MI	$Q^{AB/F}$	SSIM
TarDAL	6.35	35.48	3.13	9.91	1.49	1.82	0.42	0.71
SwinFusion	6.62	43.00	3.57	11.09	1.69	3.14	0.65	0.96
U2Fusion	6.04	29.28	2.56	9.19	1.01	2.04	0.47	0.77
SeAFusion	6.65	41.84	3.70	11.11	1.69	2.79	0.68	0.99
DATFuse	6.48	36.48	3.57	10.93	1.41	2.70	0.64	0.90
MetaFusion	6.37	39.43	3.56	12.17	1.49	1.16	0.48	0.80
DDFM	6.17	28.91	2.50	7.37	1.45	1.88	0.46	0.89
CDDFuse	6.70	43.37	3.75	11.56	1.62	**3.47**	0.69	1.00
A2RNet	6.39	37.78	2.75	8.67	1.45	2.21	0.43	0.67
EMMA	6.62	44.59	3.79	11.56	1.63	2.94	0.64	0.96
BTSFusion	6.29	33.85	**4.49**	11.82	1.37	1.58	0.49	0.79
SDCFusion	6.71	42.66	3.96	11.83	1.73	2.67	**0.71**	0.96
Ours	**6.75**	**46.01**	4.30	**12.67**	**1.74**	3.24	0.67	**1.03**

**Table 2:** Quantitative results of comparative experiments on the TNO dataset. The highest values are presented in **bold** to highlight the comparative performance, while the second-highest values are underlined.

Dataset: TNO Fusion Dataset								
Method	EN	SD	AG	SF	SCD	MI	$Q^{AB/F}$	SSIM
TarDAL	6.83	41.17	4.38	12.40	1.51	1.90	0.41	0.95
SwinFusion	6.92	41.53	4.29	10.88	1.72	1.86	0.52	0.97
U2Fusion	7.03	38.09	4.13	8.79	0.98	1.45	0.44	0.94
SeAFusion	7.13	44.95	4.91	12.06	1.72	2.02	0.50	0.99
DATFuse	6.58	29.87	3.62	9.56	1.45	2.36	0.51	0.94
MetaFusion	7.11	**48.36**	4.86	12.10	1.73	1.16	0.31	0.77
DDFM	6.81	33.34	3.13	7.82	1.72	1.56	0.40	0.97
CDDFuse	7.14	46.00	4.80	13.19	1.76	2.21	0.54	**1.03**
A2RNet	7.12	46.09	3.48	8.93	1.68	1.78	0.33	0.75
EMMA	7.18	46.75	4.67	11.17	1.66	2.14	0.48	0.97
BTSFusion	6.82	34.67	**5.78**	14.01	1.62	1.26	0.42	0.94
SDCFusion	7.09	41.58	5.16	13.09	1.72	1.79	0.53	0.97
Ours	**7.21**	47.48	5.58	**14.70**	**1.81**	**2.43**	0.47	1.01

**Table 3:** Quantitative results of comparative experiments on the RoadScene dataset. The highest values are presented in **bold** to highlight the comparative performance, while the second-highest values are underlined.

Dataset: RoadScene Fusion Dataset								
Method	EN	SD	AG	SF	SCD	MI	$Q^{AB/F}$	SSIM
TarDAL	7.07	42.97	3.87	14.16	1.38	1.80	0.45	0.90
SwinFusion	7.01	45.10	3.54	11.65	1.73	1.86	0.50	0.99
U2Fusion	7.16	38.16	5.34	11.05	1.53	1.57	0.27	0.64
SeAFusion	7.31	50.99	4.91	15.49	1.72	2.31	0.50	0.94
DATFuse	6.67	32.16	3.24	10.95	1.28	**2.63**	0.52	0.92
MetaFusion	7.07	48.83	5.71	16.45	1.63	1.56	0.40	0.82
DDFM	6.98	36.67	3.05	9.59	1.72	2.01	0.44	0.88
CDDFuse	7.41	54.55	5.80	16.14	1.80	2.34	0.52	0.96
A2RNet	7.22	51.77	3.74	12.56	1.65	2.23	0.37	0.79
EMMA	7.36	54.17	5.83	14.68	1.72	2.32	0.47	0.89
BTSFusion	6.96	35.92	5.52	16.72	1.50	1.53	0.45	0.88
SDCFusion	7.25	45.18	5.14	16.62	1.72	1.92	**0.54**	0.90
Ours	**7.46**	**55.56**	**6.86**	**17.60**	**1.86**	2.07	0.46	**1.00**

**Table 4:** Quantitative results of comparative experiments on the M3FD dataset. The highest values are presented in **bold** to highlight the comparative performance, while the second-highest values are underlined.

Dataset: M3FD Fusion Dataset								
Method	EN	SD	AG	SF	SCD	MI	$Q^{AB/F}$	SSIM
TarDAL	6.78	40.11	4.34	12.86	1.55	2.20	0.41	0.88
SwinFusion	6.81	36.01	4.74	14.24	1.57	2.15	0.60	0.99
U2Fusion	6.78	34.17	4.13	10.58	1.53	1.88	0.57	0.81
SeAFusion	6.86	35.19	4.90	14.46	1.58	2.53	0.59	0.95
DATFuse	6.41	26.08	3.48	10.67	1.30	**2.88**	0.48	0.92
MetaFusion	6.83	37.13	6.08	16.33	1.71	1.66	0.41	0.76
DDFM	6.69	30.11	3.20	9.37	1.69	2.00	0.45	0.85
CDDFuse	6.91	36.94	4.98	15.36	1.65	2.79	0.61	1.01
A2RNet	6.85	34.54	3.21	9.39	1.51	2.30	0.33	0.65
EMMA	6.95	38.38	5.43	15.69	1.51	2.41	0.59	0.90
BTSFusion	6.75	33.03	**6.63**	**17.84**	1.56	1.74	0.49	0.84
SDCFusion	6.94	36.10	5.45	16.21	1.66	2.23	0.67	0.89
Ours	**7.09**	**43.09**	6.32	16.93	**1.84**	2.62	**0.70**	**1.03**

**Table 5:** Ablation study metrics results on the MSRS Test set.

Model	EN	SD	AG	SF	SCD	MI	$Q^{AB/F}$	SSIM
w/o PCT	6.68	43.32	3.78	11.63	1.59	2.67	0.67	1.01
w/o HRM	6.71	45.18	3.53	11.87	1.57	2.86	0.57	0.81
w/o PCT and HRM	6.44	42.83	3.21	10.67	1.64	2.76	0.58	0.98
Ours	**6.75**	**46.01**	**4.30**	**12.67**	**1.74**	**3.24**	**0.67**	**1.03**

## Data Availability

The authors confirm that the data supporting this study’s findings are available within the article.

## Appendix A

This appendix provides the complete per-image metric comparisons referenced in Section 4 of the main manuscript. For each of the four benchmark datasets—MSRS, TNO, RoadScene, and M3FD—we include detailed performance curves for the proposed method (HDF-Net) and 12 representative fusion baselines.

## Appendix B

Figure A5 presents enlarged regions from representative MSRS scenes to supplement the main results. These examples highlight challenging conditions such as intense illumination and low-contrast structures. Compared with other methods, HDF-Net preserves more transparent textures and edges without introducing artifacts, confirming its advantage in handling fine-grained details under complex visual conditions.
